# Supplementary material for: The interactome of intact mitochondria by cross-linking mass spectrometry provides evidence for coexisting respiratory supercomplexes
Source: Mol Cell Proteomics. 2017 Dec 8;17(2):216–32. doi: 10.1074/mcp.RA117.000470 (PMC5795388; doi:10.1074/mcp.RA117.000470)
Supplement: Supplemental Data [file supp_RA117.000470_133922_0_supp_23978_fzffwf.zip › spectra_annotation/mito_spectra_annotation/352-1-4-1-6-1.pdf]

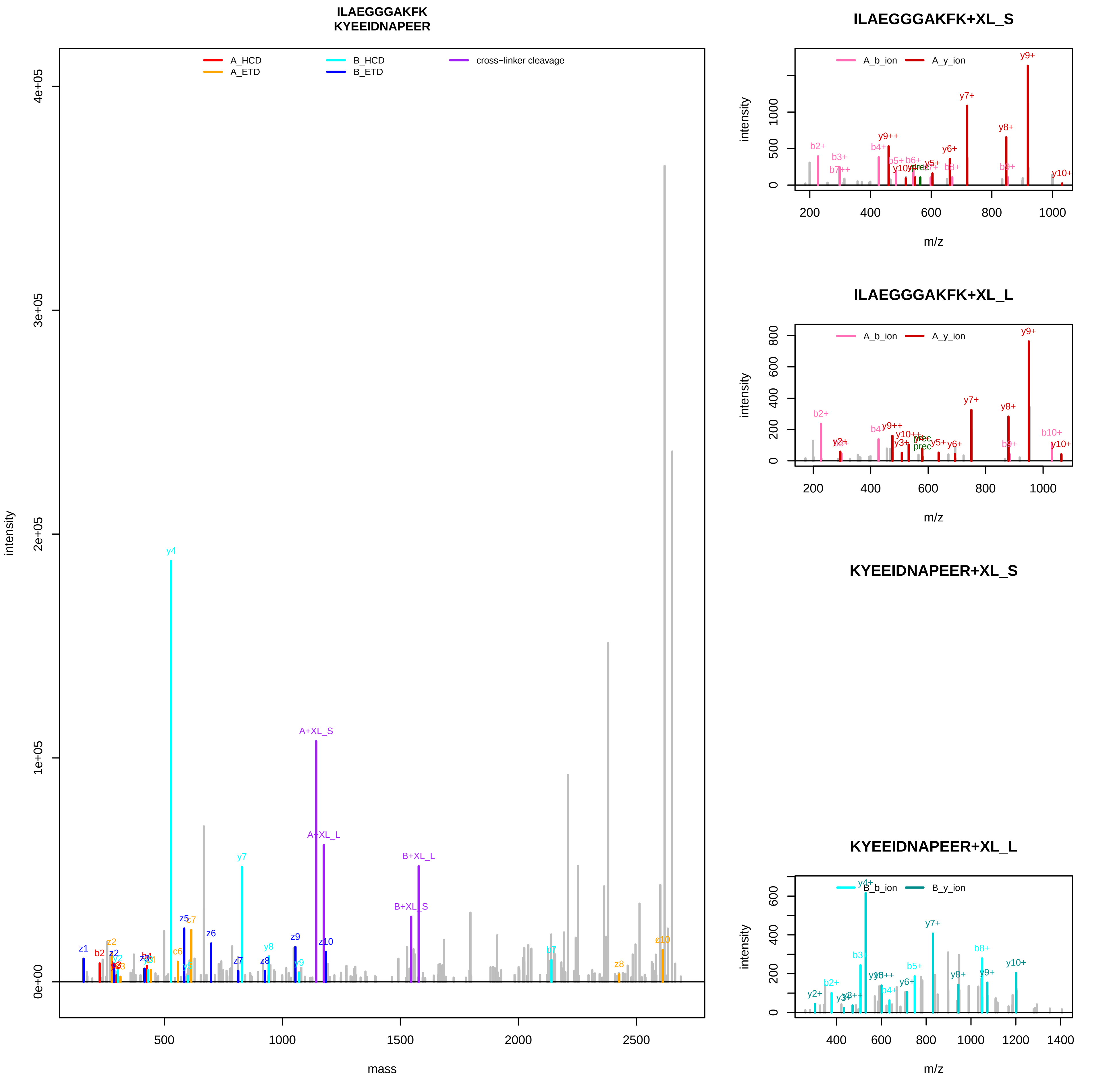

ILAEGGGAKFK
KYEEIDNAPEER
A_HCD
A_ETD
B_HCD
B_ETD
cross−linker cleavage
intensity
mass
y4
y7
A+XL_S
A+XL_L
B+XL_L
B+XL_S
z1
b2
z2
z5
c7
z6
z9
z10
z8
z7
y8
y9
c6
z10
z8
b7
ILAEGGGAKFK+XL_S
A_b_ion
A_y_ion
y9+
y7+
y8+
y9++
b2+
b3+
b4+
y6+
y5+
y10+
m/z
ILAEGGGAKFK+XL_L
b10+
KYEEIDNAPEER+XL_S
KYEEIDNAPEER+XL_L
B_b_ion
B_y_ion
y4+
y7+
b8+
y10+
b3+
b2+
y2+
b5+
y8+
y9+
y6+
